# High burden of untreated syphilis, drug resistant *Neisseria gonorrhoeae*, and other sexually transmitted infections in men with urethral discharge syndrome in Kampala, Uganda

**DOI:** 10.1186/s12879-022-07431-1

**Published:** 2022-05-07

**Authors:** Matthew M. Hamill, Annet Onzia, Tza-Huei Wang, Agnes N. Kiragga, Yu-Hsiang Hsieh, Rosalind Parkes-Ratanshi, Ethan Gough, Peter Kyambadde, Johan H. Melendez, Yukari C. Manabe

**Affiliations:** 1 Division of Infectious Disease, Johns Hopkins School of Medicine, 5200 Eastern Avenue, Mason F. Lord Center Tower, Suite 381, Baltimore, MD 21224 USA; 2 Infectious Disease Institute, Kampala, Uganda; 3 Johns Hopkins University, Baltimore, MD USA; 4 Johns Hopkins Bloomberg School of Public Health, Baltimore, MD USA; 5 Ministry of Health, National Sexually Transmitted Infections Control Program, Kampala, Uganda

## Abstract

**Objectives:**

Prompt diagnosis and treatment of sexually transmitted infections (STIs) are essential to combat the STI epidemic in resource-limited settings. We characterized the burden of 5 curable STIs chlamydia, gonorrhea, trichomoniasis, *Mycoplasma genitalium*, syphilis, and HIV infection in Ugandan men with urethritis.

**Methods:**

Participants were recruited from a gonococcal surveillance program in Kampala, Uganda. Questionnaires, penile swabs were collected and tested by nucleic acid amplification. Gonococcal isolates were tested for antimicrobial sensitivity. Sequential point-of-care tests on blood samples were used to screen for syphilis and HIV. Bivariable and multivariable multinomial logistic regression models were used to estimate odds ratios for preselected factors likely to be associated with STIs. Adherence to STI treatment guidelines were analyzed.

**Results:**

From October 2019 to November 2020, positivity (95% CI) for gonorrhea, chlamydia, trichomoniasis, and *Mycoplasma genitalium*, were 66.4% (60.1%, 72.2%), 21.7% (16.8%, 27.4%), 2.0% (0.7%, 4.9%), and 12.4% (8.7%, 17.3%) respectively. All *Neisseria gonorrhoeae* isolates were resistant to ciprofloxacin, penicillin, and tetracycline, but susceptible to extended spectrum cephalosporins and azithromycin. HIV and syphilis prevalence was 20.0% (50/250) and 10.0% (25/250), and the proportion unaware of their infection was 4.0% and 80.0% respectively. Most participants were treated per national guidelines. Multivariable analysis demonstrated significant associations between curable STI coinfections and younger age, transactional sex, but not HIV status, nor condom or alcohol use.

**Conclusions:**

STI coinfections including HIV their associated risk factors, and gonococcal AMR were common in this population. The majority with syphilis were unaware of their infection and were untreated. Transactional sex was associated with STI coinfections, and > 80% of participants received appropriate treatment.

**Supplementary Information:**

The online version contains supplementary material available at 10.1186/s12879-022-07431-1.

## Introduction

The global epidemic of sexually transmitted infections (STIs) continues unabated. There was a worldwide increase in STIs estimates between 2012 [1] and 2016 [2] from 357 to 376 million cases of four curable STIs, chlamydia, gonorrhea, syphilis and trichomoniasis. The Africa region, and Sub-Saharan Africa (SSA) particularly, has very high rates of STIs including the highest incidence rates of gonorrhea and trichomoniasis, and the highest prevalence of HIV, gonorrhea, and syphilis, and male chlamydia, and female trichomoniasis [2]. The direct sequela of STIs are grave and the implications can be particularly severe for women and their babies [3]. Furthermore, STIs enhance transmission and acquisition of HIV [4]; the risk of HIV transmission is particularly marked in the presence of more than one curable STI [5]. In a 2016 study, those with bacterial STI coinfections compared with a single infection had 11.8 and 4.1 per 100 person year incidence of HIV respectively [5]. Accurate diagnosis of STIs is important for targeted treatment, but laboratory infrastructure is frequently suboptimal in resource-limited settings (RLS), like Uganda, where syndromic case management is used for empiric algorithmic treatment of STI syndromes. The lack of specificity of syndromic management can result in missed diagnosis in the asymptomatic, as well as inappropriate treatment [6].

The prevalence of non-HIV STIs in men in SSA is poorly documented. In the most comprehensive estimate in 2019, of 130 global studies of trichomoniasis, chlamydia and gonorrhea, only 8 involved African men [2]. This is due both to donor emphasis on HIV and because men have historically been difficult to access. A 2018 World Health Organization report of male urethral discharge syndrome (UDS) demonstrated that in 2016–2017, the rates per 100,000 men aged 15–49 years were 982.9 (3.7–6133.7) in the Africa region compared to the global median, 96.7 (1.1–6133.7) [7]. UDS rates give a sense of the high burden of disease in Africa, but do not provide data on the prevalence of specific infections. We assessed the prevalence of HIV, and the 5 curable STIs: syphilis, *Chlamydia trachomatis* (CT), *Neisseria gonorrhoeae* (NG), *Trichomonas vaginalis* (TV), and *Mycoplasma genitalium* (MG) in men with UDS, nested within an existing Enhanced Gonococcal Antimicrobial Surveillance Program (EGASP) in Kampala, Uganda [8]. Additionally, we evaluated NG- antimicrobial resistance (AMR), adherence to treatment guidelines, and factors associated with STI coinfections.

## Methods

Men with self-reported symptomatic urethritis, were recruited at EGASP-participating government health centers in Kampala, Uganda where they underwent usual care clinical evaluation. The health centers, provided general healthcare and were not dedicated STI clinics. Local standards of care included the syndromic management of sexually transmitted infections (STIs) and HIV testing. Prior to joining the study, all participants were diagnosed and treated using syndromic case management as per Ugandan national guidelines [9]. Clinical care was not a component of this study. Penile-meatal swabs, blood, demographic, and behavioral data were collected. Demographic details, sexual and social behaviors, including self-reported alcohol use, self-reported transactional sex, and condom use were assessed by structured interview by trained research staff. Participants received a small transport reimbursement of Ugandan shilling 20,000 (equivalent to approximately $5–6).

### Laboratory

In Kampala, point-of-care-tests (POCTs) were performed according to the manufacturer’s instructions for treponemal antibody [Laborex RDT (Zheijing Orient Gene Biotech Co. Ltd, China)], HIV testing followed the rapid, sequential testing algorithm using the Determine (Alere, Waltham, MA), and Stat-Pak assay (Chembio, NY), with SD-Bioline (Standard Diagnostics, Giheung‐gu, Republic of Korea) as a tie breaker as required [8]. Cultures for NG were performed by EGASP in Kampala. All isolates were evaluated using disk diffusion (Kirby-Bauer test) and a subset by Etest method (BioMérieux, Marcy-l’Etoile, France) for susceptibility to ceftriaxone, cefixime, ciprofloxacin, and azithromycin [10] using Clinical and Laboratory Standards Institute (CLSI) criteria used for determining AMR. Ten milliliters (ml) of blood was collected by standard venipuncture and 1 ml serum aliquots prepared and frozen at -80 °C prior to shipping. To ensure consistency and to avoid inter-laboratory variation [11], reverse-sequence syphilis testing was conducted at Johns Hopkins University (JHU) and included a screening chemiluminescence antibody test (DiaSorin, Saluggia, Italy) with reflex to Rapid Plasma Reagin (RPR) (Cardinal Health, Dublin, Ohio) and titer. Discrepant results were adjudicated using the *Treponema pallidum* Particle Agglutination (TPPA) assay (Fujirebio, Tokyo, Japan). Penile-meatal swabs were eluted in phosphate-buffered saline, frozen at – 80 °C, and shipped to JHU for nucleic acid amplification tests (NAATs) using the Aptima CT/NG, TV, and MG assays (Hologic Inc., Marlborough, MA, USA) as previously described [12].

### Statistical analyses

Socio-demographics, clinical characteristics, and risk behaviors were described for the total study sample and by number of curable STI coinfections (none, one, and two or more) using frequencies and proportions or medians and interquartile ranges for categorical or numeric data, respectively. Differences between study participants by number of STI co-infections were determined by Fisher Exact test or Kruskal–Wallis rank sum test as appropriate. STI prevalence per mono-infection type, number of coinfections and type of coinfection was also calculated with 95% confidence intervals (CI) for the total study population. STI coinfection was defined as 2 or more curable STIs simultaneously detected in the same individual.

We also investigated factors associated with coinfection of any of the following STIs: syphilis, gonorrhea, chlamydia, trichomoniasis, and *Mycoplasma genitalium*. To determine socio-demographic, clinical or risk characteristics that explained the odds of membership in each STI coinfection category, we used bivariable multinomial logistic regression. The potential combinations of STI coinfections was too large to allow meaningful analyses, therefore the outcome was defined as a nominal variable of none, one, or two or more STIs. The number of TV positive samples was too small (n = 5) to allow constructive individual analysis. To identify participant characteristics that independently explained odds of  membership in each STI coinfection category, we fit a multivariable multinomial logistic regression model that included the following covariates: age, condom use, condomless sex with a sero-different or -unknown partner, HIV status, transactional sex, condomless sex in the past 12 months with men or women, alcohol use before sex in the past 6 months, number of partners in the past 2 months, and reported sexual activity since UDS symptom onset, based on a priori associations of these variables with STI risk [11] (Model 1). Multivariable models were refined (Model 2) by optimizing Akaike’s Information Criterion using backward stepwise selection from Model 1. All analyses were performed in RStudio version 4.0.2 software. The *nnet* package was used to implement multinomial logistic regression.

### Ethical oversight

In Uganda, the Joint Clinical Research Center at Makerere University (JC0919), and the Ugandan National Council for Science and Technology (HS455ES) approved the study. Approval was also obtained from the Johns Hopkins Institutional Review Board (IRB00215298). No study procedures were commenced until written informed consent had been obtained.

## Results

### Study population, STI prevalence

A total of 250 men with UDS, were recruited at 6 government clinics in Kampala, Uganda between October 2019 and November 2020, the study was suspended between March and July 2020 because of COVID-19 lockdown measures.

Table 1 describes the prevalence of STI mono- and co-infections in the total population. The proportion (95% CI) of participants with positive NAATs for NG, CT, TV, and MG were 66.4% (60.1%, 72.2%) (n = 164), 21.7% (16.8%, 27.4%) (n = 54), 2.0% (0.7%, 4.9%) (n = 5), and 12.4% (8.1%, 16.5%) (n = 31), respectively. Of the 164 NG NAAT-positive samples, 86.6% (n = 142) were NG culture-positive. By disk diffusion, all NG isolates were resistant to ciprofloxacin, penicillin, and tetracycline. Of 142 culture positive samples 124 (87.3%) were tested by Etest in addition to disk diffusion and were susceptible to extended spectrum cephalosporins (ESCs), and azithromycin. Cost constraints and limited supplies of Etests during the COVID-19 pandemic in Uganda meant that Etest could not be performed on all NG isolates. POC tests for syphilis were reactive in 10.0% (25/250); 80.0% (20/25) of syphilis infections were previously undiagnosed. RPR titers ranged from 1:1 to 1:1024, 28.0% (7/25) had an RPR titer of ≥ 1:8, and 30.0% (8/25) were RPR negative. All participants who reported a previous syphilis diagnosis had titers ≤ 1:1. One in 5 (50/250) POCTs for HIV were positive; 96.0% (48/50) were previously diagnosed. Overall, 84.0% of participants had at least one STI, the number, proportion (95% CI) of participants with 0, 1, 2, 3, 4, and 5 curable STIs were 40 (16.0%, 95% CI: 11.8%, 21.3%), 152 (60.8%, 95% CI: 54.4%,66.8%), 48 (19.2%, 95% CI: 14.6%,24.7%), 9 (3.6%, 95% CI: 1.8%,7.0%), 1 (0.4%, 95% CI: 0.02%,2.6%), and 0 (0.0%, 1.5%) respectively.

**Table 1:** Positivity, prevalence and 95% CI for STI mono- and co-infections in men with Urethral Discharge Syndrome in Kampala, Uganda

	Positivity	Prevalence (95% CI)
*Chlamydia trachomatis* (CT)	54	21.7% (16.8%, 27.4%)
*Neisseria gonorrhoeae* (NG)	164	66.4% (60.1%, 72.2%)
*Mycoplasma genitalium* (MG)	31	12.4% (8.7%, 17.3%)
*Trichomonas vaginalis* (TV)	5	2.0% (0.7%, 4.9%)
Syphilis	25	10.0% (6.7%, 14.6%)
HIV	50	20.0% (15.3, 25.6%)
Syphilis without HIV	9	3.5% (1.7%, 6.7%)
HIV without Syphilis	34	12.0% (8.6%, 16.5%)
HIV and Syphilis Co-infection	16	6.0% (3.6%, 9.8%)
Number and proportion with STI (including HIV)		
None	31	(12.4%, 95% CI: 8.7%, 17.3%)
1	136	(54.4%, 95% CI: 48.0%, 60.7%)
2	61	(24.4%, 95% CI: 19.3%, 30.3%)
3	18	(7.2%, 95% CI: 4.4%, 11.3%)
4	3	(1.2%, 95% CI: 0.3%, 3.8%)
5	1	(0.4%, 95% CI: 0.02%, 2.6%)
Number and proportion with Curable STIs		
None	40	(16.0%, 95% CI: 11.8%, 21.3%)
1	152	(60.8%, 95% CI: 54.4%, 66.8%)
2	48	(19.2%, 95% CI: 14.6%, 24.7%)
3	9	(3.6%, 95% CI: 1.8%, 7.0%)
4	1	(0.4%, 95% CI: 0.02%, 2.6%)
5	0	(0.0%, 95% CI: 0.0%, 1.5%)
The below prevalence counts any patient with a positive result for ALL tests listed in a row		
Only HIV	8	3.3 (1.5, 6.6)
Only NG	97	39.6 (33.5, 46.0)
Only CT	17	6.9 (4.2, 11.1)
Only MG	11	4.5 (2.4, 8.1)
Only HIV and Syphilis	2	0.8 (0.1, 3.2)
Only HIV and NG	19	7.8 (4.9, 12.0)
Only HIV and CT	1	0.41 (0.02, 2.61)
Only HIV and MG	1	0.41 (0.02, 2.61)
Only HIV and CT and NG	2	0.8 (0.1, 3.2)
Only HIV and CT and Syphilis	1	0.4 (0.0, 2.6)
Only HIV and MG and NG	2	0.8 (0.1, 3.2)
Only HIV and MG and Syphilis	1	0.4 (0.0, 2.6)
Only HIV and NG and Syphilis	6	2.5 (1.0, 5.5)
Only HIV and CT and MG and Syphilis	1	0.4 (0.0, 2.6)
Only HIV and MG and Syphilis and TV	1	0.4 (0.0, 2.6)
Only HIV and NG and Syphilis and TV	1	0.41 (0.0, 2.6)
Only HIV and CT and MG and NG and Syphilis	1	0.41 (0.0, 2.6)
Only CT and MG	3	1.2 (0.3, 3.8)
Only CT and NG	18	7.4 (4.5, 11.6)
Only CT and Syphilis	4	1.6 (0.5, 4.4)
Only CT and MG and NG	3	1.2 (0.3, 3.8)
Only CT and NG and Syphilis	1	0.4 (0.0, 2.6)
Only CT and NG and TV	2	0.8 (0.1, 3.2)
Only NG and Syphilis	4	1.6 (0.5, 4.4)
Only MG and NG	6	2.5 (1.0, 5.5)
Only MG and TV	1	0.41 (0.02, 2.61)

Table 2 describes the characteristics of study participants. The vast majority (95.2%) reported sex with women only, 0.4% of participants reported consistent or ‘always’ condom use, 61.6% reported engagement in transactional sex in the prior 6-months, and almost all (94.4%) reported condomless sex in the past 12 months with women only. Reported alcohol use was high; of the alcohol users, 56.7% used alcohol before sex, and 29.2% reported being intoxicated before sex.

**Table 2:** Demographic, behavioral, and clinical characteristics of men with Urethral Discharge Syndrome in Kampala, Uganda

	Total
N	250
Age/sexual behaviors/alcohol	
Age	24.0 [22.0, 32.0]*
Condom use	
Always	1 (0.4%)
Sometimes	149 (59.6%)
Never	100 (40.0%)
Condom use with someone of unknown or different HIV status past 6 mos, No	150 (60.5%)
Transactional sex in past 6 months, Yes	154 (61.6%)
Sex with women only	238 (95.2%)
Condomless sex past 12 mos	236 (94.4%)
Sex with both women and/or men	12 (4.8%)
Condomless sex past 12 mos	10 (4.0%)
Number of sex partners Past 2 months	2.0 [1.0,2.0]
Sexual activity since symptoms began	
Yes, with condom	10 (4.0%)
Yes, without condom	54 (21.8%)
Partner informed of symptoms	
Yes	103 (41.2%)
Planning to inform partner of diagnosis, Yes	183 (76.2%)
Alcohol use, Yes	
In past 6 mos	121 (48.4%)
Before sex in past 6 mos	68 (56.7%)
Intoxicated before sex in past 6 mos	35 (29.2%)
Symptoms	
Number of episodes of UDS in past 6 months	1.0 [1.0,2.0]*
0	6 (2.4%)
1–2	226 (90.4%)
3	18 (7.2%)
Symptom duration	
< 6 days	104 (41.6%)
≥ 6 days	146 (58.4%)

*Median [IQR], *mos* months

### Clinical, demographic, behavioral characteristics by STI co- and individual-infections

Table 3 demonstrates the distribution of 5 curable STIs comparing none, 1, and 2 or more coinfections. To further explore these associations, multinomial logistic regression was conducted and is presented in Table 4. Table 4 illustrates the multivariable multinomial logistic regression models (Model 1) comparing one vs none, and 2 or more vs none STI infections. When comparing one vs none there were significant associations with age 0.95 (0.90, 0.99), p = 0.029, and engagement in transactional sex 2.35 (1.07, 5.20), p = 0.034. Comparing ≥ 2 vs none, age remained significant 0.94 (0.89, 1.00), p = 0.050; transactional sex 2.57 (1.00, 6.64), p = 0.051 and number of partners in the prior 2 months 0.74 (0.54, 1.01), p = 0.058 were associated ≥ 2 vs no STI coinfections but did not reach statistical significance. However, none of the other factors, significant by bivariable analysis, were robust to multivariable analysis. Additional file 1: tables provides a more complete picture of Model 1. Model 2, using a backward stepwise selection from Model 1, gave very similar results (data not shown).

**Table 3:** Distributions of number of STI infections^†^ by Sociodemographics, Clinical Characteristics and Risk Behaviors in men with Urethral Discharge Syndrome in Kampala, Uganda

	Total	None	One	Two or More	p-value
N	250	40	152	58	
Positive HIV POCT	50 (20.0%)	9 (22.5%)	25 (16.4%)	16 (27.6%)	0.161
Age/sexual behaviors/alcohol					
Age	24.0 [22.0, 32.0]	27.0 [23.0, 35.3]	24.0 [22.0, 31.0]	24.0 [22.0, 33.6]	0.172
Condom use sometimes/always	150 (60.0%)	26 (65.0%)	91 (59.9%)	33 (56.9%)	0.730
Condomless sex with sero-different or -unknown partner	150 (60.5%)	25 (62.5%)	95 (62.9%)	30 (52.6%)	0.405
Transactional sex	154 (61.6%)	21 (52.5%)	97 (63.8%)	36 (62.1%)	0.411
Sex with women only	238 (95.2%)	38 (95.0%)	145 (95.4%)	55 (94.8%)	1.000
Condomless sex (past 12 mos)	236 (94.4%)	37 (92.5%)	144 (94.7%)	55 (94.8%)	0.858
Sex with women and/or men	12 (4.8%)	2 (5.0%)	7 (4.6%)	3 (5.2%)	1.000
Condomless sex (past 12 mos)	10 (4.0%)	2 (5.0%)	5 (3.3%)	3 (5.2%)	0.724
Number partners past 2 mos	2.0 [1.0,2.0]	1.0 [1.0,3.0]	2.0 [1.0,2.0]	2.0 [1.0,2.0]	0.313
Sexually active since symptoms with condom	10 (4.0%)	3 (7.5%)	5 (3.3%)	2 (3.4%)	0.367
Sexually active since symptoms without condom	54 (21.8%)	11 (27.5%)	31 (20.7%)	12 (20.7%)	0.532
Partner informed of symptoms yes	103 (41.2%)	13 (32.5%)	65 (42.8%)	25 (43.1%)	0.509
Planning to inform partner of diagnosis yes	183 (76.2%)	24 (64.9%)	114 (77.6%)	45 (80.4%)	0.197
Alcohol use past 6 mos yes	121 (48.4%)	19 (47.5%)	69 (45.4%)	33 (56.9%)	0.335
Alcohol use before sex past 6 mos	103 (44.4%)	19 (47.5%)	56 (40.3%)	28 (52.8%)	0.274
Symptoms					
Symptom duration ≥ 6 days	146 (58.4%)	25 (62.5%)	90 (59.2%)	31 (53.4%)	0.673
Discharge and/or pain yes	250 (100.0%)	40 (100.0%)	152 (100.0%)	58 (100.0%)	1.000
Burning on urination yes	234 (93.6%)	36 (90.0%)	143 (94.1%)	55 (94.8%)	0.584

^†^NG or CT or TV or MG or Syphilis, *mos* months

**Table 4:** Multivariable Logistic Regression Model of number of curable STI infections, sociodemographic and clinical characteristics or risk behaviors in men with Urethral Discharge Syndrome in Kampala, Uganda

	One vs. none (n = 137)		Two or more vs. none (n = 53)	
	OR (95% CI)	p-value	OR (95% CI)	p-value
Age in years	0.95 (0.90, 0.99)	**0.029**	0.94 (0.89, 1.00)	**0.050**
Living with HIV: Yes vs. No	1.92 (0.55, 6.69)	0.305	2.63 (0.65,10.54)	0.173
Condom use sometimes/always: Yes vs. No	0.80 (0.36, 1.76)	0.575	0.77 (0.31, 1.93)	0.580
Transactional: Yes vs. No	2.35 (1.07, 5.20)	**0.034**	2.57 (1.00, 6.64)	0.051
Condomless sex in past 12mos men or women: Yes vs. No	0.84 (0.09, 8.01)	0.880	2.63 (0.23,30.44)	0.439
Alcohol before sex past 6 mos: Yes vs. No^1^	0.81 (0.37, 1.74)	0.587	1.33 (0.54, 3.24)	0.533
Number partners past 2 mos	0.94 (0.83, 1.07)	0.351	0.74 (0.54,1.01)	0.058
Sexually active since symptoms: Yes with condom vs. No^2^	0.33 (0.07, 1.62)	0.172	0.43 (0.06, 2.97)	0.389
Sexually active since symptoms: Yes without condom vs. No^2^	0.86 (0.35, 2.12)	0.744	0.91 (0.31, 2.61)	0.854

*mos* months

^1^17 reported “unknown” and 1 declined to answer and were excluded; ^2^2 declined to answer and were excluded; N = 230; Bold type indicates a p-value of < 0.05

### Antimicrobial treatment

Adherence to Ugandan guidelines for the treatment of UDS, consisting of cefixime plus doxycycline, was high. Overall 84.0% received or were prescribed cefixime 400 mg as a single dose, and 93.2% doxycycline 100 mg twice daily for 7 days.

## Discussion

One key finding was the very high prevalence of gonorrhea, and > 12% MG positivity among men with UDS in Kampala, adding to the data on etiological infections associated with UDS in men in Africa. The low condom usage, high reported alcohol consumption and intoxication before sex, reluctance to inform sex partners about UDS symptoms, and a majority reporting engagement in transactional sex sets the scene for high-level, sustained STI transmission in this population, onward transmission to sex partners, and increased risk for HIV transmission and acquisition [4]. In the multivariable regression, younger age predicted diagnosis of at least 2 curable STIs suggesting that these men were at particular high risk for STI likely compounded by engagement in transactional sex and more sexual partners. The cross-sectional design prevented assessment of the sequential relationship between curable STIs and HIV; in other settings, prior syphilis was a risk factor for HIV acquisition [13]. However, in this sample of men HIV prevalence was higher than syphilis; this may be explained by high prevalence of high-risk sexual behaviors in those already living with HIV mediated by risk compensation associated with taking ART.

Adherence to the 2016 Ugandan guidelines for the treatment of UDS was very high (at least 84%), similar to the findings of a US study in 2016 where 81.3% received recommended NG treatment [14]. Based on contemporary Ugandan NG-AMR data, with very low resistance to ESC [8, 15], it can be expected that the vast majority of genital NG, and CT infections would be eradicated by currently recommended UDS dual therapy. Conversely, MG is unlikely to be eradicated by doxycycline, in a Swedish study only 38% of men achieved cure with doxycycline therapy [16]. We were unable to ascertain microbiological cure in the participants to compare those who eradicated infection and those who did not. Larger scale MG prevalence data are warranted in the Ugandan setting to establish the role of MG in UDS, prevalence of resistance mutations, and to inform STI treatment guidelines. In addition, future studies should also involve extragenital testing to understand the burden of extragenital STI in those with UDS. In this sample 4.8% of men reported having sex with other men, increasing the risk for extragenital STIs; this is likely an underestimate given the social desirability bias resulting from criminalization and stigma associated with same-sex sexual activity in Uganda.

Ongoing global funding efforts for HIV diagnosis and treatment, and efforts such as the 90–90–90 goals drives high ascertainment of HIV infection as shown in this sexually active cohort where 96% of men with HIV were aware of their status. HIV (20.0%) and syphilis (10.0%) positivity was high, compared to the national averages in men of 3.9% [3.3–4.2] [17] and 2.0% [18], respectively. Unlike HIV, most participants with syphilis were unaware of their diagnosis. Given readily available syphilis-, and dual HIV/syphilis-POCTs [19], this missed opportunity for syphilis diagnosis and treatment requires urgent attention.

Other work in SSA have explored the etiological causes of UDS. A Zimbabwean study [20] of 200 symptomatic men described STI positivity similar to those we present, however the proportion with > 2 infections was lower at 1% compared with 23.2% seen in this study. This may be explained by a higher prevalence of sexual risk behaviors or higher background STI prevalence in this Ugandan study. As expected, STI positivity seen in these symptomatic men is much higher than from general male population surveys in Uganda [21]. The participants in this study were an unselected group of adult males with UDS attending government health centers in Kampala, not STI clinic attendees where STI prevalence is likely to be higher still, so some generalizability to non-STI-focused health centers in Kampala is reasonable. Other, more contemporary, work has focused on the high burden of STIs in female sex workers and men who have sex with men; in the latter, the prevalence of non-HIV STI was 13.5% [22]. In a 2015 study of motorcycle taxi drivers, of 683 men, HIV prevalence was 7.5%, syphilis, CT, and NG positivity was 6.1%, 1.1% and 1.2%, respectively [23]. Future work should include those without symptomatic urethritis to compare prevalence of STI and associated risk factors.

STI and HIV risk was high in the men who participated in this study, as condom use was sporadic, and a large proportion of men engaged in behaviors that increase the risk for STI transmission. Younger age, and engaging in transactional sex were significantly associated with STIs in multivariable analysis. Other associations anticipated to be predictive of STI coinfection, such as HIV status, condom use and alcohol use were not associated with STI coinfections in the multivariable analysis. This unanticipated finding may reflect the very high community burden of curable STI and overall low condom use. Almost three in five participants waited for at least 6 days before attending a clinic suggesting barriers to access to clinical care. We did not assess the proportion with a new HIV diagnosis who achieved viral suppression on ART after diagnosis, nor measure CD4 count so were unable to assess immune compromise in those with HIV. However, those with HIV sought UDS care sooner, perhaps indicating greater familiarity with healthcare systems; it is possible that impaired immunity associated with HIV resulted in greater symptom burden; such hypotheses require further investigation.

STI can increase HIV infection risk [4], “classical” STIs act as what Cohen calls an ‘amplifying factor’ for increased HIV transmission via mucosal inflammation and increased concentration of HIV in genital secretions [24]. In this group of young men, the high proportion already living with HIV and active STI suggest missed prevention opportunities. To build on the successes of Uganda’s 90–90–90 progress, young men who engage in transactional sex, and other high-risk sexual behaviors should be identified for prevention interventions before they acquire HIV and other STIs or at their first episode of UDS. Behavior change interventions are perennially challenging [25], but have been shown to be effective [26] so merit ongoing efforts. In particular interventions to promote consistent condom use with all partners, including transactional and non-transactional partnerships, are required [27].

The strengths of this study include gold standard molecular diagnosis of four UDS-associated STIs in a CAP-certified laboratory. The high prevalence of HIV allowed us to better define the association of risk behaviors and STI in a population largely aware of their HIV status. Future studies are required to explore post-treatment outcomes of men treated for UDS, including persistent or new curable STIs, evolving AMR, incident HIV infection, and partner notification interventions.

## Conclusion

Bacterial STIs were very common in this group of largely heterosexual men with UDS. Syphilis prevalence was 5-times greater than that seen in the general male population and the majority of cases were previously undiagnosed. These findings support the urgent introduction of POCTs for syphilis and immediate treatment in the setting of UDS. The associations with younger age, transactional sex, and greater number of recent sex partners suggest an ongoing reservoir of STI in those exchanging sex, further work is required to quantify this. The study demonstrates the need for continued NG AMR surveillance, and a MG surveillance program to track MG burden and evolution of AMR. We show how effectively existing surveillance programs can be leveraged to collect data on other STIs. Local clinical services that provide UDS treatment were demonstrated to serve a population with a high burden of STIs, although the majority were currently not living with HIV. Such clinics may present an ideal opportunity for HIV prevention interventions including condom education and provision, and HIV PrEP in those at risk.

## Supplementary Information

**Additional file 1.** Multivariable logistic regression model of number of curable STI infections, sociodemographic and clinical characteristics or risk behaviors in men with urethral discharge syndrome in Kampala, Uganda with I) None, and II) Two or more as the referent.

## Data Availability

The datasets used and/or analysed during the current study available from the corresponding author on reasonable request.
