# Supplementary material for: High burden of untreated syphilis, drug resistant Neisseria gonorrhoeae, and other sexually transmitted infections in men with urethral discharge syndrome in Kampala, Uganda
Source: BMC Infect Dis. 2022 May 7;22:440. doi: 10.1186/s12879-022-07431-1 (PMC9077641; doi:10.1186/s12879-022-07431-1)
Supplement: Supplementary file 1 — Additional file 1. Multivariable logistic regression model of number of curable STI infections, sociodemographic and clinical characteristics or risk behaviors in men with urethral discharge syndrome in Kampala, Uganda with I) None, and II) Two or more as the referent. [file 12879_2022_7431_MOESM1_ESM.docx]

**Additional table S1. Multivariable Logistic Regression Model of number of curable STI infections, sociodemographic and clinical characteristics or risk behaviors** **in men with Urethral Discharge Syndrome in Kampala, Uganda, with None as the referent.**

|  | **None vs. One**  **(n=40)** | | **Two or More vs. One**  **(n=53)** | |
| --- | --- | --- | --- | --- |
|  | **OR (95%CI)** | **P-value** | **OR (95%CI)** | **P-value** |
| Age in years | 1.06(1.01,1.11) | **0.029** | 1.00(0.95,1.05) | 0.874 |
| Living with HIV: Yes vs. No | 0.52(0.15,1.81) | 0.306 | 1.37(0.51,3.69) | 0.536 |
| Condom use sometimes/always: Yes vs. No | 1.25(0.57,2.77) | 0.575 | 0.97(0.49,1.91) | 0.927 |
| Transactional: Yes vs. No | 0.42(0.19,0.94) | **0.034** | 1.09(0.52,2.32) | 0.817 |
| Condomless sex in past 12mos men or women: Yes vs. No | 1.19(0.12,11.35) | 0.879 | 3.13(0.53,18.40) | 0.207 |
| Alcohol before sex past 6 mos: Yes vs. No^1^ | 1.24(0.57,2.67) | 0.588 | 1.64(0.83,3.24) | 0.151 |
| Number partners past 2 mos | 1.06(0.94,1.21) | 0.351 | 0.79(0.59,1.06) | 0.112 |
| Sexually active since symptoms: Yes with condom vs. No^2^ | 3.06(0.62,15.23) | 0.172 | 1.30(0.21,8.03) | 0.775 |
| Sexually active since symptoms: Yes without condom vs. No^2^ | 1.16(0.47,2.86) | 0.744 | 1.05(0.46,2.40) | 0.904 |

mos=months

^1^17 reported “unknown” and 1 declined to answer and were excluded; ^2^2 declined to answer and were excluded; N=230

**Additional table S2. Multivariable Logistic Regression Model of number of curable STI infections, sociodemographic and clinical characteristics or risk behaviors** **in men with Urethral Discharge Syndrome in Kampala, Uganda, with Two or more as the referent**

|  | **None vs. Two or More**  **(n=40)** | | **One vs. Two or More**  **(n=137)** | |
| --- | --- | --- | --- | --- |
|  | **OR (95%CI)** | **P-value** | **OR (95%CI)** | **P-value** |
| Age in years | 1.06(1.00,1.13) | **0.05** | 1.00(0.96,1.05) | 0.874 |
| Living with HIV: Yes vs. No | 0.38(0.09,1.53) | 0.173 | 0.73(0.27,1.97) | 0.536 |
| Condom use sometimes/always: Yes vs. No | 1.29(0.52,3.23) | 0.580 | 1.03(0.52,2.04) | 0.927 |
| Transactional: Yes vs. No | 0.39(0.15,1.00) | 0.051 | 0.92(0.43,1.94) | 0.817 |
| Condomless sex in past 12mos men or women: Yes vs. No | 0.38(0.03,4.41) | 0.440 | 0.32(0.05,1.88) | 0.207 |
| Alcohol before sex past 6 mos: Yes vs. No^1^ | 0.75(0.31,1.84) | 0.533 | 0.61(0.31,1.20) | 0.151 |
| Number partners past 2 mos | 1.35(0.99,1.84) | 0.058 | 1.27(0.95,1.70) | 0.112 |
| Sexually active since symptoms: Yes with condom vs. No^2^ | 2.35(0.34,16.40) | 0.389 | 0.77(0.12,4.73) | 0.775 |
| Sexually active since symptoms: Yes without condom vs. No^2^ | 1.10(0.38,3.18) | 0.854 | 0.95(0.42,2.17) | 0.904 |

mos=months

^1^17 reported “unknown” and 1 declined to answer and were excluded; ^2^2 declined to answer and were excluded; N=230
